# Training Attentional Control in Infancy

**DOI:** 10.1016/j.cub.2011.08.004

**Published:** 2011-09-27

**Authors:** Sam Wass, Kaska Porayska-Pomsta, Mark H. Johnson

**Affiliations:** 1 Centre for Brain and Cognitive Development, Department of Psychological Sciences, Birkbeck, University of London, London WC1E 7HX, UK; 2 London Knowledge Lab, Institute of Education, London WC1N 3QS, UK

## Abstract

Several recent studies have reported that cognitive training in adults does not lead to generalized performance improvements [1, 2], whereas many studies with younger participants (children 4 years and older) have reported distal transfer [3, 4]. This is consistent with convergent evidence [5–8] for greater neural and behavioral plasticity earlier in development. We used gaze-contingent paradigms to train 11-month-old infants on a battery of attentional control tasks. Relative to an active control group, and following only a relatively short training period, posttraining assessments revealed improvements in cognitive control and sustained attention, reduced saccadic reaction times, and reduced latencies to disengage visual attention. Trend changes were also observed in spontaneous looking behavior during free play, but no change was found in working memory. The amount of training correlated with the degree of improvement on some measures. These findings are to our knowledge the first demonstration of distal transfer following attentional control training in infancy. Given the longitudinal relationships identified between early attentional control and learning in academic settings [9, 10], and the causal role that impaired control of attention may play in disrupting learning in several disorders [11–14], the current results open a number of avenues for future work.

## Highlights

► Infants were trained using a battery of gaze-contingent attentional control tasks ► Training led to improved cognitive control, sustained attention, and reduced RTs ► Training resulted in trend changes in looking behavior during free play ► Training had no effect on working memory

## Results

We trained infants using a battery of gaze-contingent computer tasks targeting attentional control (see Experimental Procedures). Task 1 (butterfly) featured a target that “flew” only while the infant looked directly at it, with distractors presented in the periphery of the visual field; task 2 (stars) and task 4 (elephant) featured search for changing targets with distractors of varying salience; and task 3 (windows) targeted working memory for objects embedded in scenes of varying complexity.

### Study Structure

Forty-two typically developing 11-month-old infants took part in five lab visits over 15 days. A pre- and posttesting battery was administered at the first and last visits. Between these visits, the trained group (n = 21) completed on average 77 min of training; the control group (n = 21) completed the same number and duration of lab visits but viewed infant-appropriate television clips and animations for an equivalent amount of time.

### Training Results

**Figure 1:**
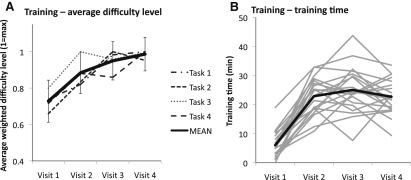
Results from Training (A) Training: average difficulty level. The longest unbroken instance of each training task per session was identified, and the average difficulty level of each task was calculated (see Supplemental Experimental Procedures). Average difficulty level at visit 4 was normalized to 1, to allow comparison between the degrees of improvement at the different tasks. Error bars represent standard errors. (B) Training: training time. Gray lines show per-session training times for individual participants; the thick black line shows the average. The large change between visit 1 and visits 2–4 is because visit 1 was conducted immediately following the pretest assessment battery, so infants had already conducted circa 90 min of testing.

Outcome measures were used for each task to determine task difficulty level during training. The difficulty level changed adaptively during training in response to participants' performance, according to procedures outlined in the Supplemental Experimental Procedures available online. Repeated-measures analyses of variance identified significant increases in the average difficulty level across the training sessions for task 2 (stars) [F(1,39) = 9.90, p < 0.001], task 3 (windows) [F(1.58,14.17) = 4.85, p = 0.03], and task 4 (elephant) [F(1,18) = 4.70, p = 0.014] (see Figure 1A).

### Pre-post Test Results

Table S1 shows the raw and baseline-corrected results for all of the pre-post measures. We conducted analyses of covariance (ANCOVA) with the factor group (trained versus control), post-test scores as the dependent variable, and pretest scores as the covariate. This is equivalent to an ANCOVA on the difference scores with pretest as a covariate [15]. Values of Cohen's d were calculated from the marginal means.

#### A: Cognitive Control

**Figure 2:**
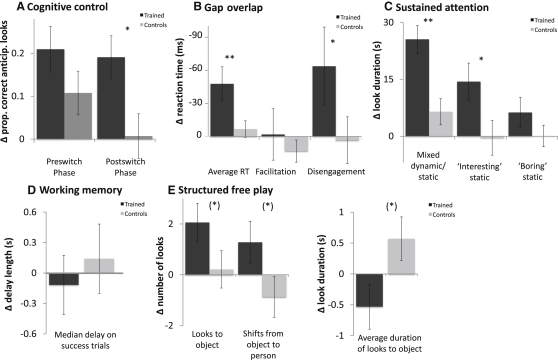
Results of Pre-post Assessments All plots show Δ, the change in performance (post − pre, baseline corrected) in the trained and control groups. Error bars represent standard errors. Asterisks indicate significance of ANCOVA analyses as described in Results: ^∗^p < 0.05; ^∗∗^p < 0.01; (^∗^)p < 0.10. (A) Cognitive control. Graph shows proportion of correct anticipatory looks in the preswitch (initial rule learning) and postswitch (unlearning one rule and learning another) phases. (B) Gap overlap. “Average RT” is the average of the three conditions that we administered (gap, baseline, and overlap). “Facilitation” shows the facilitation effect, and “disengagement” shows disengagement latencies. Because the valence of the observed changes in the task was negative, −Δ values are presented for ease of comparison. (C) Sustained attention. “Mixed dynamic/static” shows the results of experiment 1, which measured looking behavior toward a mixture of dynamic and static stimuli. “Interesting static” and “boring static” show the results of experiment 2, which measured looking behavior toward “interesting” and “boring” static images. (D) Working memory. Graph shows median delay length for trials followed by a correct response. (E) Structured free play. “Looks to object” shows number of separate looks to the target objects. “Shifts from object to person” shows number of attention shifts from looking at the objects to looking at either the experimenter or caregiver. “Average duration of looks to object” shows the average duration of looks toward the target objects.

**Figure 3:**
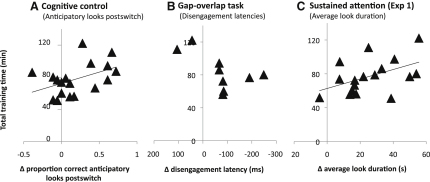
Results of Pre-post Assessments: Selected Scatter Plots from the Trained Group Showing Amount of Training Time against Change in Performance at Posttesting The y axes show the amount of total training time (in minutes) that each participant received. The x axes show trained infants' change in performance (post − pre) on three measures; in (B), −Δ values are presented for ease of comparison. For all three graphs, a position to the right of the y axis indicates improved performance posttraining. The regression lines indicate the significant bivariate correlations (see Results) observed between training time and outcome measures in (A) and (C).

This was an anticipatory looking task in two phases—the preswitch phase tested initial rule learning, and the postswitch phase tested ability to inhibit a previously learned rule while acquiring a new rule [16]. An ANCOVA indicated more correct anticipatory looks at posttesting in the trained group relative to the controls in the postswitch [F(1,34) = 6.57, p = 0.015, Cohen's d = 0.69] but not the preswitch phase [F(1,34) = 1.91, p = 0.18] (see Figure 2A). A bivariate correlation was identified between improvement at the postswitch phase and the amount of training time [r(18) = 0.449, p (one-tailed) = 0.031], suggesting that more training was associated with greater improvement at posttesting (see Figure 3A).

#### B: Gap Overlap

This task assessed components of visual attention [12]. Three trial types were administered—gap, baseline, and overlap (see Supplemental Experimental Procedures). From these, we calculated disengagement latencies (time to disengage visual attention from one target in order to fixate another one, defined as overlap − baseline) and facilitation effects (cueing effect of a temporal gap preceding the onset of the peripheral stimulus, defined as baseline − gap) (following [12]). Final analysis showed that training led to reduced reaction times on gap [F(1,23) = 5.19, p = 0.032, Cohen's d = 0.60] and overlap [F(1,23) = 10.6, p = 0.003, Cohen's d = 0.83] and nonsignificant reductions on baseline [F(1,22) = 2.22, p = 0.15, Cohen's d = 0.53]. Averaging the three conditions revealed globally reduced reaction times posttraining [F(1,19) = 12.02, p = 0.003, Cohen's d = 1.06]. Although the facilitation effect did not change significantly after training, the disengagement effect did [F(1,22) = 6.81, p = 0.016, Cohen's d = 0.68] (see Figure 2B). For the whole sample without exclusion of outliers, the average reaction-time effect was still significant, but other effects (e.g., disengagement latencies) were nonsignificant trends (see Table S2 and Figure S2).

#### C: Sustained Attention

Experiment 1 assessed infants' looking behavior toward a mix of novel dynamic and nondynamic images [17]. An increase was found posttraining on average look duration [F(1,33) = 14.39, p = 0.001, Cohen's d = 1.03]. A bivariate correlation was observed between increase in average look duration and amount of training time [r(17) = 0.477, p (one-tailed) = 0.026] (see Figure 3C). Experiment 2 [18] assessed looking behavior toward “interesting” and “boring” static images. A significant effect of training was identified for duration of the longest unbroken look to the “interesting” [F(1,36) = 4.19, p = 0.048, Cohen's d = 0.65] but not to the “boring” stimuli [F(1,36) = 1.75, p = 0.194] (see Figure 2C).

#### D: Working Memory

This task assessed the ability to generate saccades toward objects following a variable delay length [19]. No significant changes were observed following training [F(1,37) = 3.564, p = 0.175] (see Figure 2D).

#### E: Structured Free Play

Infants sat in front of a puppet theater and were presented with a series of novel objects [20], and their spontaneous viewing behavior was analyzed. Trend effects of training were found for increased number of attentional shifts from object to person (experimenter or caregiver) [F(1,34) = 3.33, p = 0.077, Cohen's d = 0.54], shorter average duration of looks to the objects [F(1,34) = 4.1, p = 0.051, Cohen's d = 0.58], and increased number of separate looks to the objects [F(1,34) = 3.01, p = 0.092, Cohen's d = 0.44] (see Figure 2E).

### Discussion

We administered a battery of gaze-contingent attentional control tasks that targeted maintaining an online goal ([21]; cf. [22]), inhibition [23], and search for a changing target [24], as well as visuospatial working memory [25, 26]. Relative to a matched active control group, we found that a relatively short training period led to improved cognitive control and sustained attention, as well as reduced average saccadic reaction times. Attention disengagement latencies were also significantly reduced, but only after exclusion of outliers. Trend (p < 0.1) changes were found in spontaneous looking behavior during free play (trend toward more and shorter looks toward the novel object and toward more attentional shifts from object to person). Our assessment of working memory found no evidence of improvements following training.

A number of general factors could potentially have influenced the training effects observed. First, could the improvements in sustained attention have arisen because the contingent nature of the training stimuli left the trained group generally more motivated to orient toward the screen than controls? The fact that our sustained attention experiments identified larger increases in looking time toward “interesting” than toward “boring” images seemingly precludes this, indicating a degree of selectivity in the effect (see Figure 2C; cf. [18]). Second, could the increased cognitive control and reduced saccadic latencies that we found have been caused by improved sustained attention? Trial-by-trial analyses of the cognitive control task (Figure S1) show that between-group differences were most evident in trials 4–6 postswitch and actually decreased during trials 7–9, which counts against this possibility. Third, we found considerable differences between the (quasirandomly assigned) trained and control groups at pretesting on certain measures (e.g., the free play task and the second sustained attention experiment) (see Table S1). Could these differences at pretesting have influenced the training effects we found? The ANCOVA analysis we used is considered adequately to account for this [15]. Fourth, it is possible that the infant-appropriate sustained attention assessments that we used—looking time to novel stimuli [17, 18]—may measure something different from the techniques used to assess sustained attention in older children (e.g., continuous performance task [11]).

Although novel within the infant literature, our results are consistent with work on attentional control and executive components in older children. Improved cognitive control and sustained attention as well as reduced reaction times have been reported following related forms of executive control training (e.g., [4, 24, 25]). Globally increased reaction-time latencies have also been associated with decreased general executive factors and inattentive behavior [27]. Given the links between working memory and attentional control [26], our failure to find a training effect on working memory is surprising; this may be because working memory, although detectable [19], is weak at this early age. A full investigation of the factor analytic structure underlying the training effects that we found is a goal for future work (see e.g. [28]).

We also observed trend effects of training during free play with toys. These results are consistent with previously reported correlations between attentional control and spontaneous looking behavior during infancy (e.g., [29]). Using a similar task, Kannass and Oakes [20] found that more spontaneous attentional reorienting at 9 months (more frequent, shorter looks) correlated with better language development at 31 months. It is interesting that the trained group in the present study showed fewer, longer looks on the sustained attention task (where there was one interesting target in a room from which other distractions had been removed) and more, shorter looks in another (a room with a variety of interesting targets and people present). One possibility is that increased attentional control may make the allocation of attention more flexible, depending on context (see [18]).

### Neural and Behavioral Plasticity Early in Development

Many studies with adults [1, 2] and older adults [2, 30] have failed to show transfer of improvements following training (although see [22, 31, 32]). In contrast, and consistent with convergent evidence of increased neural and behavioral plasticity earlier in development [5–8], a number of studies with children aged 4 years and over have shown transfer of training improvements following cognitive training (e.g., [4, 24, 25, 26, 33]).

The ability to control attention may be critically required for the subsequent acquisition of a range of other skills—for example, executive attention may be an important “tool for learning” in language acquisition [13]. In atypical development, early abnormalities in attentional control may lead to cascade-like disruptions over developmental time [14]. For example, problems with disengaging visual attention in autism spectrum disorders may impair learning in social situations ([12], but see [34]). Impaired attentional control may also disrupt subsequent learning in infants showing hyperactivity-impulsivity [35] and those born preterm [36, 37], as well as those from low-socioeconomic status (SES) backgrounds [10]. This suggests the desirability of very early interventions when behavioral plasticity may be greater, and before subsequent catastrophic developmental cascades have taken place [14].

To our knowledge, this is the first report of distal transfer of training effects following cognitive training in participants younger than 4 years old [3, 4]. (Jankowski et al. [38] successfully manipulated infants' allocation of visual attention by shining lights at a display, although they did not assess transfer to other tasks; see also [39].) In this regard, it is striking that we found changes following briefer training periods than those used by other studies (77 min versus 375 min for 4- to 5-year-olds in Thorell et al. [4]). Further work is required to assess whether this is because infant brains are more plastic and more readily amenable to training or because eye-gaze contingent training is more immersive in comparison with the point-and-click computer interface used by other groups.

The most significant limitation to this study is that we only assessed changes shortly (circa 15 days) after the commencement of training. Although other studies have shown that improvements following training persist at medium-term follow-up [26], it is possible that the relatively more plastic infant brain is more readily trainable but that these improvements dissipate more rapidly. Further work is needed to assess this question and to assess the impact of longer periods of training.

## Experimental Procedures

Participants were 42 (21 trained [T], 21 control [C]) typically developing infants. Average age at visit 1 was 339 (standard deviation 9.2) days for the trained group, and 335 (9.2) days for the control group. Gender ratios were 14 male/7 female (T) and 12 male/9 female (C). Two participants (1 T, 1 C) failed to complete the study. Parents were blinded to group assignment and to the specific aims of the study. The study involved five lab visits over 15 days (T = 16 [2.2], C = 15 [1.5]). Visit 1 was pretesting followed immediately by training session 1; visits 2, 3, and 4 were dedicated training visits; and visit 5 was posttesting, which was identical to the pretesting battery. The eye tracker used was a 50 Hz Tobii 1750 (1024 × 768 pixels, monitor subtending 24° × 29°); stimuli were presented using the Talk2Tobii toolbox [40] and custom-written MATLAB scripts. Training stimuli were presented until infants became fidgety or distressed. On average, 77 (19.1) min of training was completed per participant. Control sessions were conducted in the same room, with the same experimenters, and using the same eye tracker as the training sessions and had the same duration and spacing (yoked to a trained participant). Instead of training, control participants viewed a selection of infant-friendly television clips and still images.

### Training Stimuli

#### Training Protocol

Four gaze-contingent training tasks were presented in rotation at each visit. The tasks were based on extensive piloting and had different difficulty levels that changed adaptively depending on performance (see Supplemental Experimental Procedures). Each task was presented until the infant became inattentive, at which point they went to the next task or took a break.

#### Task 1 (Butterfly)

A target (a butterfly, subtending 6°) was presented on the screen. When the infant fixated the target, the butterfly “flew” across the screen, and distractors (a house, a tree, clouds; 5°–15°) scrolled in the opposite direction. When the infant looked to one of the distractors, the distractors disappeared and only the target, now static, remained on screen. On refixating the target, it recommenced moving and the distractors reappeared and continued scrolling. The salience of the distractors changed adaptively, including faster, larger, and more densely packed objects. This task targeted selective/focused attention and interference resolution.

#### Task 2 (Stars)

One of five possible targets (each a cartoon character in a brightly colored star; 6°) was presented on screen together with eight distractors (smaller stars, planets, clouds; 4°–8°) against a detailed still image as background. If the infant fixated the target within 3000 ms, he or she received an animation as a reward. The target changed from trial to trial. The salience of the distractors changed adaptively (including moving, spinning, and shrinking distractors). This task targeted search for a changing target and ignoring distractors.

#### Task 3 (Windows)

When the infant fixated the target (an animal in a window, subtending 7°), an animation showed the target disappearing into one of several windows that were then covered with curtains. A fixation target (a flower; 4.5°) appeared elsewhere on the screen and rotated when the infant looked at it. After a delay period, the fixation target disappeared. If the infant looked back to the window behind which the target had disappeared, he or she received an animation as a reward. The number of windows, the salience of the distractors, and the length of the delay changed adaptively. This task trained visuospatial working memory and required acting on stored information about objects embedded in complex scenes.

#### Task 4 (Elephant)

A target (an elephant; 4.5°–8°) was presented with one or more distractor items of the same size. When the infant looked at the target, he or she received an animation as a reward. The same target was then re-presented with one or more other distractors. If the infant successfully fixated the target within the time limit, he or she received an animation as a reward; if not, the trial reset. The number of distractors varied adaptively. After 28 trials, the target changed. This task targeted task switching, visual search, and inhibiting the urge to look at distractors.

### Pre-post Tests

Six pre-post tests were administered to infants in the trained and control groups pre- and posttraining. These were based on previously published infant-appropriate assessments of cognitive control (modeled on [16]), saccadic reaction times (gap-overlap task, modeled on [12]), sustained attention (two tasks, modeled on [17 and [18]), working memory (modeled on [19]), and spontaneous attentional orienting during free play with toys (modeled on [20]). The exact methods employed are described in Supplemental Experimental Procedures.

## Supplemental Information

Document S1. Two Tables, Two Figures, and Supplemental Experimental Procedures
