# Supplementary material for: Training Attentional Control in Infancy
Source: Curr Biol. 2011 Sep 27;21(18):1543–7. doi: 10.1016/j.cub.2011.08.004 (PMC3202624; doi:10.1016/j.cub.2011.08.004)
Supplement: Document S1. Two Tables, Two Figures, and Supplemental Experimental Procedures [file mmc1.pdf]

Current Biology, Volume *21*

Supplemental Information

Training Attentional Control in Infancy

Sam Wass, Kaska Porayska-Pomsta, and Mark H. Johnson

Supplemental Inventory

Table S1. Raw and Marginal Means for Pre-post Assessments

Table S2. Complete Means and Dropout Rates for the Gap-Overlap Task

Figure S1. Trial-by-Trial Data of the Cognitive Control Task

Figure S2. Box Plot for the Gap-Overlap Task

Supplemental Experimental Procedures

Pre-post tests. Descriptions of the methods used in the pre-post tests.
Adaptive change criteria for training stimuli. Descriptions of the criteria for adaptive change within the training stimuli.

Supplemental References

Table S1. Raw and Marginal Means for Pre-post Assessments

	Means (StErr)				Marginal means (StErr)				
	Trained		Control		Trained		Control		Sig.
	Pre-test	Post-test	Pre-test	Post-test	Pre-test	Post-test	Pre-test	Post-test	
**A Cognitive control:** pre-switch (proportion correct anticipatory looks)	0.41 (0.06)	0.61 (0.07)	0.35 (0.07)	0.47 (0.06)	0.38 (0.6)	0.59 (0.05)	0.38 (0.6)	0.49 (0.05)	
**A Cognitive control:** post-switch (proportion correct anticipatory looks)	0.31 (0.05)	0.50 (0.06)	0.31 (0.04)	0.32 (0.05)	0.31 (0.05)	0.50 (0.05)	0.31 (0.05)	0.32 (0.05)	*
**B Gap-overlap task:** gap condition (RT (msecs))(a)	265 (5)	246 (3)	272 (3)	260 (3)	268 (5)	249 (3)	268 (5)	260 (3)	*
**B Gap-overlap task:** baseline condition (RT (msecs))(a)	355 (9)	322 (3)	362 (6)	351 (8)	356 (7)	330(4)	356 (7)	346 (8)	
**B Gap-overlap task:** overlap condition (RT (msecs))(a)	697 (25)	569 (16)	650 (21)	649 (18)	671 (23)	578 (16)	671 (23)	661 (18)	**
**C Sustained attention (Exp 1):** mixed dynamic/static - average look duration to target (secs)	22.7 (3)	51.6 (4)	24.2 (4)	30.9 (4)	24.9 (3)	50.5 (4)	24.9 (3)	31.4 (4)	**
**C Sustained attention (Exp 2):** 'interesting' static - peak look duration (secs)	30.5 (4.5)	47.5 (5.4)	52.5 (6.0)	49.7 (6.8)	41.8 (4.8)	56.2 (5.0)	41.8 (4.8)	41.4 (5)	*
**C Sustained attention (Exp 2):** 'boring' static - peak look duration (secs)	19.6 (3.2)	25.8 (4.0)	21.6 (3.7)	21.4 (3.7)	20.7 (3.2)	26.9 (3.2)	20.7 (3.2)	20.8 (3.2)	
**D Working memory:** median delay on success trials (secs)	2.84 (0.3)	2.26 (0.3)	2.38 (0.3)	2.87 (0.3)	2.62 (0.3)	2.50 (0.3)	2.62 (0.3)	2.76 (0.3)	
**E Structured free play:** number of looks to object	13.8 (1)	16.6 (0.9)	15.9 (0.7)	15.4 (0.7)	14.9 (0.7)	16.9 (0.8)	14.9 (0.7)	15.1 (0.7)	(*)
**E Structured free play:** number of attentional shifts from object to person	7.0 (0.7)	8.9 (0.8)	9.4 (0.9)	8.0 (1.0)	8.2 (0.6)	9.5 (0.8)	8.2 (0.6)	7.4 (0.8)	(*)
**E Structured free play:** average look duration to object (secs)	7.6 (0.6)	6.6 (0.5)	5.1 (0.4)	6.1 (0.4)	6.3 (0.4)	5.8 (0.4)	6.3 (0.4)	6.9 (0.4)	(*)

Note. The final column (sig) refers to significance on ANCOVAs, as described in the results section. (*) - p<0.1, * - p<0.05, ** - p<0.01

[a] - see also supp. Table 2

Table S2. Complete Means and Dropout Rates for the Gap-Overlap Task

	Means (StErr) - N subjects			
	Trained		Control	
	Pre-test	Post-test	Pre-test	Post-test
**Average RT across all 3 conditions in ms**				
Complete data	456 (15) - 20	382 (10) - 20	430 (6) - 21	422 (10) - 21
Incomplete subjects (<12 trials)	497 (5) - 4	325 (39) - 3	438 (6) - 4	425 (48) - 4
Just subjects with >12 trials	446 (13) - 16	392 (9) - 17	428 (8) - 17	421 (9) - 17
After exclusion of outliers	433 (11) - 14	373 (6) - 12	428 (8) - 17	419 (8) - 16
**Average RT for Gap condition in ms**				
Complete data	266 (5) - 20	252 (6) - 20	274 (4) - 21	268 (7) - 21
Incomplete subjects (<12 trials)	271 (19) - 4	271 (32) - 3	285 (11) - 4	291 (35) - 4
Just subjects with >12 trials	265 (5) - 16	249 (4) - 17	272 (3) - 17	263 (4) - 17
With outliers excluded	265 (5) - 16	246 (3) - 16	272 (3) - 17	260 (3) - 16
**Average RT for Baseline condition in ms**				
Complete data	374 (12) - 20	335 (7) - 20	363 (5) - 21	356 (8) - 21
Incomplete subjects (<12 trials)	368 (21) - 4	359 (32) - 3	367 (8) - 4	376 (28) - 4
Just subjects with >12 trials	376 (16) - 16	331 (6) - 17	362 (6) - 17	351 (8) -17
With outliers excluded	355 (9) - 14	322 (3) - 14	362 (6) - 17	351 (8) - 17
**Average RT for Overlap condition in ms**				
Complete data	728 (37) - 20	588 (21) - 19	652 (18) - 21	641 (22) - 21
Incomplete subjects (<12 trials)	851 (156) - 4	519 (11) - 2	662 (25) - 4	608 (90) - 4
Just subjects with >12 trials	697 (25) - 16	596 (23) - 17	650 (21) - 17	649 (18) - 17
With outliers excluded	697 (25) - 16	569 (16) - 15	650 (21) - 17	649 (18) - 17

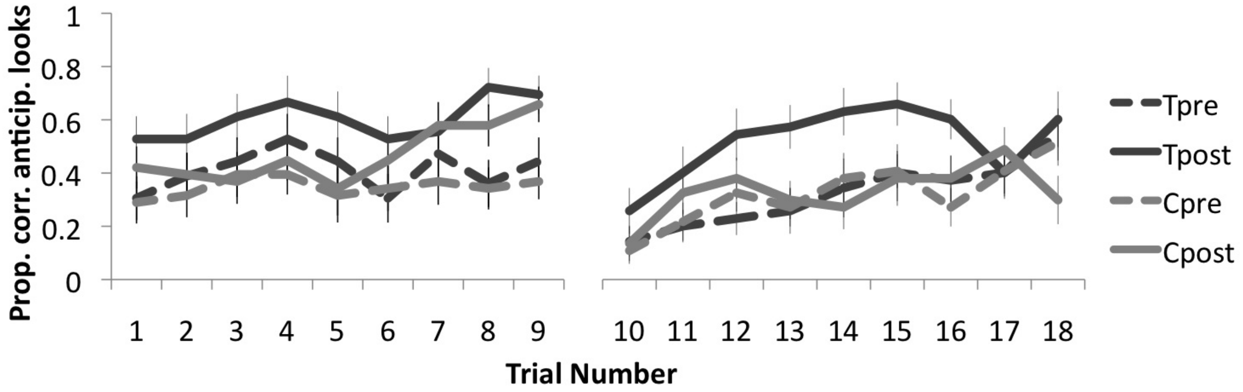

Figure S1. Trial-by-Trial Data of the Cognitive Control Task

The visual reward switched sides between trials 9 and 10: trials 1-9 are the *pre-switch phase* and trials 10-18 are the *post-switch phase*. The y-axis shows the proportion of correct anticipatory looks. Lines indicate standard errors.

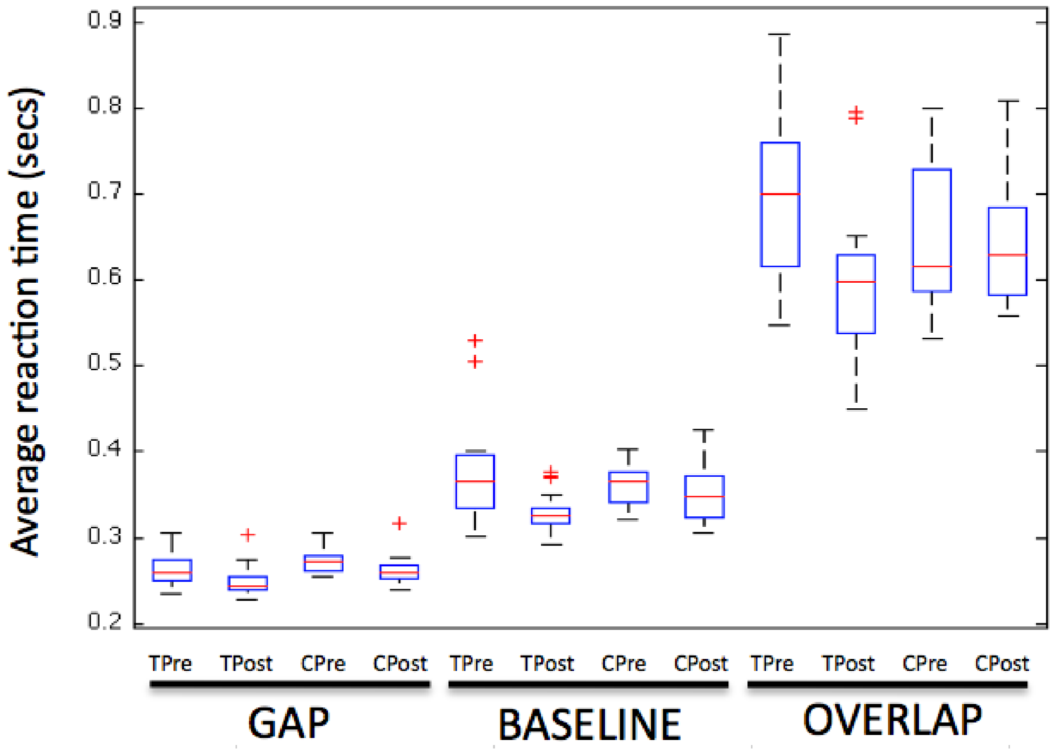

Figure S2. Box Plot for the Gap-Overlap Task

Showing the outliers that were excluded according to the criteria described in the Supplemental Experimental Procedures.

Supplemental Experimental Procedures

Pre-post Tests

Pre-post tests were administered identically before and after training. The tests were segmented into blocks and presented interleaved in a randomized order. Resting state EEG was also recorded, the results of which will be published at a later date.

A. Cognitive Control **(Modeled on [1])**

After fixating a central target (a cartoon flower subtending 4.5˚), the trial commenced following a 300 ms delay. Two blank rectangles (10.8˚ x 9˚) were presented left and right, concurrently with an auditory stimulus for 2000 ms (the anticipatory window). A visual reward (lasting 4000 ms) then appeared on one side (in either the left or right rectangle) for 9 trials in a row (the pre-switch phase) before swapping sides for the next 9 trials (the post-switch phase). If the participant correctly anticipated the presentation of the reward (defined as a saccade beginning between 300 and 2300 ms after trial onset and subject to a minimum look duration of 400 ms), then the visual reward stimulus appeared immediately. The outcome measure was proportion of correct anticipatory looks. Two blocks of 18 trials were presented. Three participants (2T, 1C) did not complete all blocks at pre- and post-testing.

B. Gap/Overlap Task **(Modeled on [2])**

After fixating a central target (CT, a cartoon flower, 4.5˚), following a variable ISI (see below) a lateral target (LT, a cartoon cloud, 3˚) was presented to the left or right; when the participant fixated the LT they received a brief audiovisual reward. Three conditions were presented: Gap - CT disappears 200 ms before LT appears; Baseline - CT disappears concurrently with LT appearance; Overlap - CT remains onscreen with LT appearance. This task was presented in two blocks. Within each block, the three conditions were presented in a pseudo-randomised order, until enough valid trials had been collected (12 usable trials per condition) or the infant became inattentive. The reaction time (RT) was the time elapsed between LT appearance and the reported position of gaze leaving the central fixation area (a 9˚ box around the CT). Reaction times less than 200 and greater than 1200 ms were excluded [2].

Data from one participant (1T) were lost due to experimenter error. The data were refined as in previous protocols [2]. First, data were excluded from a number of participants (18%) who had failed to provide 12 valid trials per condition; the number of participants that were excluded at this stage is consistent across groups, and data that were excluded at this stage show similar training effects to the included data (see Table S2). Second, a number of outliers (5%) were also excluded using the >1.5 IQR criterion (see Figure S2) [3]. Table S2 shows the group means and standard errors at each stage of processing.

C. Sustained Attention – Experiment 1 **(Modeled on [4])**

A series of objects/faces were presented: head shots of actors (single and in groups) reciting nursery rhymes, still images of actors' faces, shots of toys and birds, accompanied by background music. Individual stimuli were presented for 4 or 8 seconds; between stimuli, a blank screen and brief auditory stimulus were presented. If the participant was still looking at

the screen, it progressed to the next stimulus; if not, attention-getter sounds were repeated until the target re-fixated the target. In total, 200 seconds of material was presented in 3 x 65 second blocks. Infants' looking behavior was coded from a camera on top of the monitor. Gaze was coded in 1-second bins, as either looking at the stimulus presentation area or not. Total percentage looking time and the length of each unbroken look to the target were calculated. Looks were excluded if the total duration of the look (e.g. away from and back to the target) was under 1 second. All tapes were coded blind. 25% of the videos were double coded with an inter-rater agreement of 0.94 on looking versus not looking. Data from some participants were unavailable (4T, 2C) due to equipment or experimenter error.

C. Sustained Attention – Experiment 2 **(Modeled on [5])**
4 different still images were presented, in two blocks of two at different stages of the testing protocol. Two of these images were 'interesting' (ie attractive, detailed images (of flowers and fish)) and the other two were 'boring' (ie low-detail, monochrome outlines of a diamond and a cross). Trials commenced once the subject had fixated a central target. Trials ended when the subject had looked away from the screen for 1 second or more, or following a 15 second time-out. Following the end of a trial, a fixation target and brief auditory stimulus (<1s) were presented. If the subject fixated the target, the next trial started immediately; if not, a sequence of different fixation targets and auditory attention getters was repeated. Stimuli were re-presented until: i) two successive looks were less than 50% of the longest unbroken look so far, ii) eight successive looks had taken place without reaching criterion, or iii) the total presentation length exceeded 120 seconds [6]. Background music was playing during this task, which was identical at pre- and post-testing.

D. Working Memory **(Modeled on [7])**
After fixating a central target (CT), two doors (12˚x 5˚) appeared on the left and right; after 1000 ms, two arrows (each 8˚x 5˚) appeared laterally above and below one of the doors for 600 ms, after which the CT re-appeared and a timer was started. When the participant had fixated the CT for a variable delay period (see below), it disappeared and the two doors remained. If participants looked at the cued door within 1500 ms, an animated reward appeared for 2000 ms; if they looked at the uncued door, the trial was reset. The delay period started at 300 ms, increasing by 600 ms for correct responses and decreasing by 450 ms for incorrect. The first two trials in each block were 'show' trials - the reward appeared regardless of looking behavior. Two blocks of 12 trials per block were presented. To prevent fatigue, a short movie clip was presented mid-way through each block. The outcome measure was median delay period for trials followed by a correct response. Data from one participant (1T) were lost due to experimenter error.

E. Structured Free Play **(Modeled on [8])**
Testing was conducted in a puppet theatre behind which experimenter and the camera were visible. Infants sat on their caregiver's lap. Five unfamiliar objects were presented consecutively in randomized order for 30 seconds each. Infants' viewing behavior was videoed and coded *post hoc* in 1-second bins for whether the infant was looking at: i) object, ii) experimenter, iii) caregiver, iv) curtains/stage/camera. Sections where the object was not on the stage (because

the infant had knocked or thrown it off) were excluded. Attentional shifts from object to person were coded as looks from the object to either the caregiver or experimenter. Data were triple coded. All three coders were blinded as to group membership. Inter-rater agreement was 84.6% between coders 1 and 2, and 86.6% between coders 2 and 3, overall inter-rater agreement was 85.6%. Coder 1 coded 75%, coder 2 60% and coder 3 35%. Data from some participants (3T, 1C) were unavailable due to equipment or experimenter error during recording.

Criteria for Adaptive Change within Training Stimuli

The Experimental Procedures section in the main text contains descriptions of the training stimuli. All the training stimuli changed adaptively during training, according to criteria that were set following an extensive piloting period. Our aims were: i) to keep infants at their maximum difficulty threshold to make the training as effective as possible; ii) to keep the task varied to maintain participant interest.

Task 1 (Butterfly)

The target moved from left to right only when the infant was looking at it. Once the target had crossed from the left to the right of the screen the percentage of the total trial time for which the participant had been looking at the butterfly was calculated. If this was above 40%, an internal performance counter increased one point. If this counter was above three (i.e. if the participant had completed a total of three or more screens at >40%), then rarer, high-salience distractors started to appear. If the counter was above ten then they moved on to a higher difficulty level, with a completely different set of distractors (bigger and more closely packed). At the higher difficulty level, overlapping distractors passed in front of the butterfly, temporarily occluding it, whereas at lower difficulty levels overlapping distractors passed behind the butterfly.

Task 2 (Stars)

Seven different difficulty levels were designed – at lower difficulty levels the eight distractors were smaller, static, identical to each other and dissimilar to the targets; at higher difficulty levels they were more varied, moving and more brightly colored. Once the infant had located five targets, the average search time per target was calculated. If the average search time per target was less than five seconds, the participant progressed up a difficulty level; if it was greater than twelve seconds, they moved down a level. If the participant had stayed at the same difficulty level at the two previous occasions on which it had been adjusted, the difficulty moved up or down a level at random in order to prevent the task becoming repetitious.

Task 3 (Windows)

The task had two different adaptive measures - firstly, the length of time that the central fixation target (the flower) had to be fixated before it disappeared (i.e. the delay length), and secondly the difficulty level - the number of windows present on screen, the presence or absence of background distractors, and the complexity of the visual array in which the windows were presented. The delay length increased or decreased in 200 ms increments for every correct and incorrect trial. The difficulty level was re-assessed every three trials - if the average

success rate over the previous six trials was greater than 80%, the participant moved up a difficulty level; if less than 20%, they moved down a difficulty level.

Task 4 (Elephant)

A success trial was defined as whether the infant looked to the target within a set time period following presentation of the array. The infant's performance was evaluated every four trials. If the participant had no fail trials within the last block of four (i.e. no trials in which they failed to find the target within the allotted time period), they moved up a difficulty level and the number of distractors presented increased. If there had been more than two fail trials over the last block of eight trials then the number of distractors decreased. Change of target (from elephant to chicken) was nonadaptive, and took place every 28 trials (i.e. approximately every 2 minutes of game-play). When three or more distractors were present (i.e. for infants who were performing at a relatively high level), the reward target for the previous block of 28 trials was presented as one of the distractors (a conflict trial); when fewer distractors were present, only novel distractors were presented (nonconflict).

Supplemental References

1. Kovacs, A. M. & Mehler, J. (2009) Cognitive gains in 7-month-old bilingual infants. Proceedings of the National Academy of Sciences of the United States of America, 106(16), 6556-6560.
2. Elsabbagh, M., Volein, A., Holmboe, K., Tucker, L., Csibra, G., Baron-Cohen, S., Bolton, P., et al. (2009) Visual orienting in the early broader autism phenotype: disengagement and facilitation. Journal of Child Psychology and Psychiatry, 50(5), 637-642.
3. McGill, R., Tukey, J.W. & Larsen, W.A. (1978) Variations of box plots. American Statistician 32: 12-16.
4. Shaddy, D. J. & Colombo, J. (2004) Developmental changes in infant attention to dynamic and static stimuli. Infancy, 5(3), 355-365.
5. Courage, M. L., Reynolds, G. D. & Richards, J. E. (2006) Infants' attention to patterned stimuli: Developmental change from 3 to 12 months of age. Child Development, 77(3), 680-695.
6. Colombo, J. & Mitchell, D. W. (2009) Infant visual habituation. Neurobiology of Learning and Memory 92(2): 225-234.
7. Gilmore, R. O. & Johnson, M. H. (1995) Working Memory in Infancy – 6-month-olds performance on 2 versions of the oculomotor delayed-response task. Journal of Experimental Child Psychology, 59(3), 397-418.
8. Kannass, K. N. & Oakes, L. M. (2008) The development of attention and its relations to language in infancy and toddlerhood. Journal of Cognition and Development, 9(2), 222-246.
